# No Time to Fold: Intrinsically Disordered Microproteins in Action

**DOI:** 10.1021/acs.biochem.6c00280

**Published:** 2026-06-05

**Authors:** Tianyu Chen, Kevin Cao, Thomas F. Martinez

**Affiliations:** † Department of Pharmaceutical Sciences, University of California, Irvine, Irvine, California 92617, United States; ‡ Department of Biological Chemistry, University of California, Irvine, Irvine, California 92617, United States; § Chao Family Comprehensive Cancer Center, University of California, Irvine, Irvine, California 92617, United States

**Keywords:** microprotein, small open reading frame, intrinsically disordered protein

## Abstract

Advances in genomics, proteomics, and bioinformatics have uncovered the existence of thousands of translated small open reading frames less than 100–150 codons in length that encode microproteins. In addition to their diminutive size, microproteins are also often predicted to be intrinsically disordered based on their enrichment in disordered-promoting amino acids. Microproteins have since been found to regulate diverse cellular processes, including DNA repair, mRNA decay, mitochondrial metabolism, and ribosome biogenesis, among others. While only a small fraction of microproteins have been functionally characterized, many examples have been found to act as regulators of larger proteins and protein complexes in ways similar to annotated intrinsically disordered proteins (IDPs). In this review, we summarize the functions and mechanisms of several disordered microproteins while exploring the approaches used to study their disordered nature, their regulation by post-translational modifications, and potential strategies to therapeutically target them in disease. These examples underscore how investigations of disordered microproteins deepen our understanding of how biological processes are regulated and emphasize how close collaboration between the microprotein and IDP fields can enhance these efforts.

## Graphical abstract

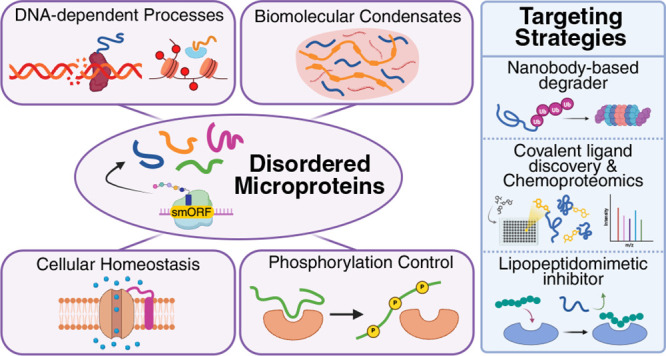

## Introduction

Microproteins are a previously neglected class of proteins encoded by small open reading frames (smORFs) less than 100–150 codons in length.^1,2^ Owing to the development of Ribo-seq^3^ and advances in mass spectrometry-based proteomics,^4,5^ we now know that thousands of smORFs are translated to produce microproteins in prokaryotes^6,7^ and eukaryotes^8–12^ alike. These smORFs are found within regions of the transcriptome presumed to be noncoding, including 5′- and 3′-untranslated regions (UTRs), long noncoding RNAs (lncRNAs), and overlapping CDS regions in alternative reading frames (Figure 1). Microproteins function in critical cellular processes, including DNA repair,^13,14^ mRNA decay,^15,16^ and calcium regulation,^17^ among others. Microproteins generally carry out these diverse activities by binding to and regulating larger proteins and protein complexes. While only a small fraction of microproteins have been functionally characterized, some patterns have emerged with regards to how they interact with other proteins and how these interactions are regulated.

**Figure 1:**
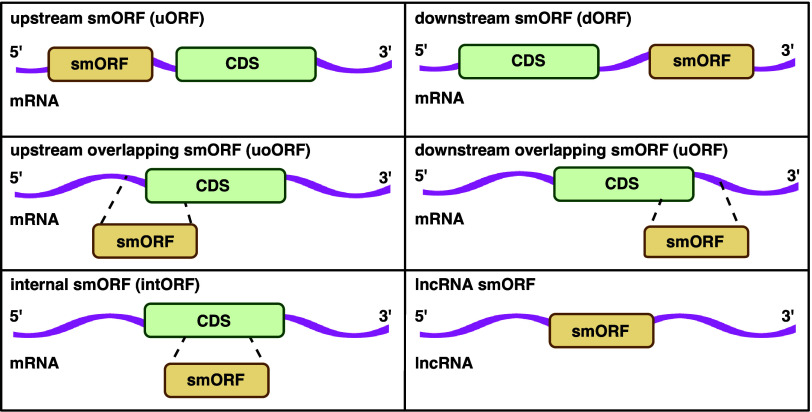
Microprotein-coding smORFs are encoded in regions on transcripts previously thought to be noncoding. Microprotein-coding smORFs can be found within 5′-UTRs, 3′-UTRs, overlapping or within coding DNA sequences (CDS) in alternative reading frames, and on long noncoding RNAs (lncRNAs). Figure created using BioRender.com.

It is now apparent that microproteins are not only inherently small, but that many characterized examples also lack ordered structures. Several studies have noted that the amino acid usage of microproteins differs from that of larger canonical proteins. Microproteins in both human^9,10^ and mouse^18^ show consistent enrichment in alanine, glycine, proline, and arginine and depletion of isoleucine, asparagine, and tyrosine on average compared to annotated proteins. This composition is similar to that of intrinsically disordered proteins (IDPs) or intrinsically disordered regions (IDRs) within annotated proteins,^19–21^ suggesting that a large fraction of microproteins are also highly disordered.^22^ This shared feature helps inform how many microproteins impact cellular and organismal physiology and suggests that the assays and tools developed to study IDPs can also apply well to microproteins.

IDPs and IDRs are abundant among annotated proteins, with recent estimates suggesting that at least 30% of human proteins contain IDRs.^23^ IDPs play key roles in cellular processes such as cell signaling, genomic and transcriptional regulation, and subcellular or structural organization.^24–28^ Instead of adopting a single stable conformation, IDPs exist as dynamic ensembles that can flex and reshape to interact with multiple partners.^29,30^ IDRs are also frequent targets of post-translational modifications (PTMs), such as phosphorylation, which can quickly alter their charge, shape, and binding preferences.^30–33^ Many IDRs contain short linear motifs (SLiMs), tiny binding segments that enable transient, selective interactions critical for signaling networks.^30,34^ IDRs can also engage in multivalent interactions that drive liquid–liquid phase separation (LLPS), giving rise to biomolecular condensates (BMC) like stress granules, P-bodies, and nucleoli.^30,35,36^ Given these diverse roles of IDPs and the disordered nature of microproteins, it is unsurprising that several characterized microproteins have also been found to act in these processes (Table 1). In the following sections, we discuss representative examples of both well-studied and more recently discovered intrinsically disordered microproteins involved in genomic regulation, BMC dynamics, and membrane-associated signaling and stress responses (Figure 2). Based on these examples, we also explore their regulation by phosphorylation, put forth potential approaches for developing targeted therapeutics against disease-relevant disordered microproteins, and highlight common effective strategies to characterize the biophysical properties of disordered microproteins and their interactions.

**Table 1:** Examples of Intrinsically Disordered Microproteins and Their Protein Interactors

microprotein	gene	amino acid length	function	protein interactor	evidence for disordered regions
CYREN	*CYREN*	157/69/102	DNA double-strand break (DSB) repair	Ku70/Ku80	NMR, CD, HDX-MS
EMBOW	*SCRIB*	120	chromatin regulation and mitotic spindle assembly	WDR5	inferred from absence of nonbinding sequence in X-ray crystal structure
NBDY	*NBDY*	68	P-body assembly and RNA turnover	EDC4/DCP1A	NMR, computational disorder prediction
p14^ARF^	*CDKN2A*	54	p53-dependent stress response	NPM1/MDM2	NMR, SANS
NISM	*HDAC5*	36	nucleolar structure and rRNA synthesis regulator	DHX9	computational disorder prediction
PLN	*PLN*	52	calcium homeostasis	SERCA/Hax-1	NMR, EPR, electron microscopy, DARR, MD simulations
SHMOOSE	*MT-ND5*	58	mitochondrial energy metabolism	IMMT	computational disorder prediction
Noxa	*PMAIP1*	54	pro-apoptotic regulator	MCL-1	CD, EPR, thermal denaturation assay, computational disorder prediction.

**Figure 2:**
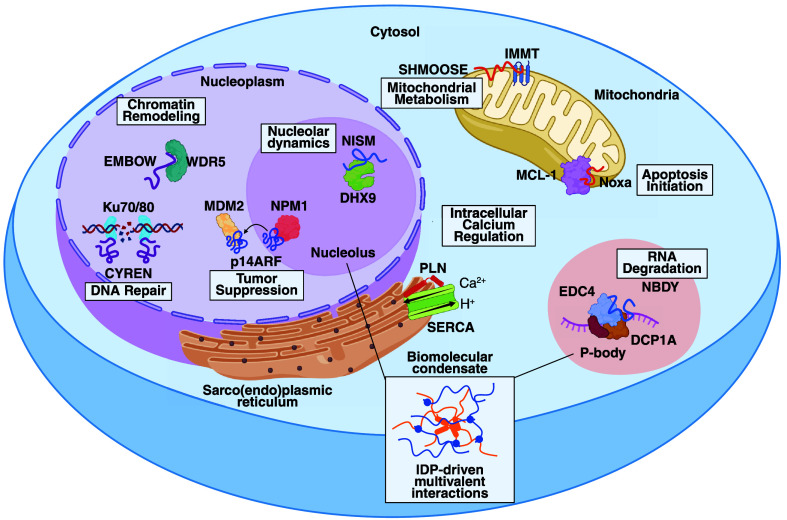
Intrinsically disordered microproteins function in a variety of cellular processes. Examples of disordered microproteins that function in different processes and subcellular compartments are depicted. Figure created using BioRender.com.

## Disordered Microproteins in DNA-Dependent Processes

### Cell-Cycle Regulator of Nonhomologous End-Joining (CYREN)

CYREN, originally named Modulator of Retroviral Infection (MRI), was first identified as a 157 amino acid protein using a genetic screen for host proteins that resist retroviral infection.^37^ Subsequent proteogenomic and biochemical analyses revealed that *CYREN* encodes multiple protein isoforms generated from distinct transcripts. Three isoforms have been characterized to date: the full-length 157 amino-acid form (CYREN-1), a short 69 amino-acid microprotein that shares its N-terminus with CYREN-1 (CYREN-2), and a 102 amino acid truncated form of CYREN-1 (CYREN-3).^13^ CYREN-1 and -2 localize to the nucleus and interact with the Ku70/Ku80 heterodimer, core components of the nonhomologous end-joining (NHEJ) machinery, which is the primary responder for repairing DNA double-stranded breaks (DSB).^13^ CYREN-1 and -2 were shown to function as inhibitors of classical NHEJ during the S and G2 phases of the cell cycle through its interaction with the Ku complex.^14^ This activity thus promotes error-free repair by homologous recombination during cell cycle phases when sister chromatids are available. In addition, a more recent study found that CYREN has a role in mediating No-indel end-joining repair of blunt DSBs as a backup factor to XLF.^38^ This study also suggested that CYREN’s inhibition of classical NHEJ during S/G2 phases is due to the presence of overhangs at the end of telomeres.

Biophysical characterization of recombinant CYREN-1 revealed that it is largely intrinsically disordered.^39,40^ Circular dichroism (CD) and nuclear magnetic resonance (NMR) analyses showed minimal secondary-structure content and narrow chemical-shift dispersion, consistent with a conformationally dynamic ensemble.^40^ Furthermore, hydrogen–deuterium exchange mass spectrometry (HDX-MS) confirmed that CYREN-1 lacks stable secondary structure, showing widespread deuterium incorporation across the sequence except for its N-terminus. The N-terminal region of CYREN-1 also showed some potential for alpha helical formation by CD in the presence of the crowding agent trifluoroethanol. Similarly, Xie et al. found CYREN-1 to be largely disordered based on NMR but also observed some alpha helical character for CYREN-1 by CD but not for CYREN-2.^39^ Altogether, these studies demonstrate that CYREN-1 and -2 are largely disordered.

CYREN-1 has also been characterized as an adaptor protein based on the presence of conserved short motifs, including an N-terminal Ku-binding motif (KBM) and a C-terminal XLF-like motif (XLM), as well as a central nonconserved disordered region that can mediate additional interactions.^40^ Together, these motifs and disordered regions are what enable recruitment of NHEJ factors, including DNA-PKcs, XLF, PAXX, and XRCC4. The C-terminal XLF-like motif (XLM) also mediates interactions with DNA damage response (DDR) proteins, including ATM and MRN. More recently, high-resolution interaction mapping revealed additional interaction motifs for CYREN1. Sequence analysis of CYREN1 and coimmunoprecipitation experiments revealed that the C-terminal region contains a SLiM (residues 148–154) that interacts with the spliceosome component SF3B1, suggesting that CYREN may also participate in splicing.^39^ Purified CYREN-1 has also been found to forms dimer and higher order oligomers in solution, and therefore may nucleate large DNA repair assemblies in cells.^39,40^ These studies demonstrate how a small disordered microprotein can facilitate complex activities by enabling interactions with multiple proteins.

### Endogenous Microprotein Binder of WDR5 (EMBOW)

EMBOW, also known as alt-SCRIB or oSCRIB, is a 120 amino acid human microprotein encoded by an upstream overlapping open reading frame (uoORF) on *SCRIB*. It was identified by proteogenomic approaches in multiple human cancer cell lines.^41,42^ Functionally, EMBOW acts both in cis, where its translation reduces downstream SCRIB translation, and in trans via its interaction with WDR5.^42,43^ EMBOW binds to the WIN site of WDR5, a conserved interaction surface used by multiple WDR5 partners, and modulates WDR5 interaction with other proteins during the cell cycle.^43^ Specifically, EMBOW’s interaction with WDR5 prevents its interaction with KMT2A and promotes its binding to KIF2A and correct mitotic spindle assembly. Structural analysis of the EMBOW-WDR5 complex by X-ray crystallography showed that binding to WDR5 is mediated almost exclusively by the N-terminal six residues of EMBOW, which bind to the WIN site of WDR5.^44,45^ The remainder of the sequence does not contribute to the structured interface in the crystal, suggesting that most of EMBOW is intrinsically disordered.^44^ Direct biophysical evidence of EMBOW’s disorder have not yet been reported, leaving open questions about its conformational ensemble and the potential roles of the disordered region.

These two examples highlight how disordered microproteins can regulate DNA-dependent processes such as DNA repair and mitosis like many canonical IDPs.^46^ Similarly, they suggest that additional examples may be uncovered by identifying cell cycle-regulated microproteins.

## Disordered Microproteins in Biomolecular Condensates

### Nonannotated P-Body Dissociating Polypeptide (NoBody/NBDY)

NBDY is a 68 amino acid microprotein that was discovered through proteogenomic analyses of K562, HEK293T, and MDA-MB-231 cells.^15^ Functionally, NBDY was shown to localize to cytoplasmic BMCs known as mRNA processing bodies, or P-bodies. P-bodies serve as a site for translationally repressed RNAs and mRNA decay factors, and thus enable mRNA decay,^47^ though P-bodies are not absolutely required for mRNA decay to occur.^48^ Within P-bodies, NBDY physically interacts with components of the mRNA decapping complex, specifically EDC4 and DCP1A.^15,16^ Overexpression of NBDY was found to disrupt P-bodies, while its inhibition increases the number of P-bodies. These activities enable NBDY to function as regulator of global RNA stability and P-body formation.^16^

Structural studies have demonstrated that NBDY is intrinsically disordered. For example, ^1^H–^15^N Heteronuclear Single Quantum Coherence (HSQC) NMR spectra show a narrow signal dispersion in the ^1^H dimension, indicating a relatively uniform chemical environment for each residue.^49,50^ NBDY’s disordered structure underlies its ability to engage in multivalent electrostatic interactions with both protein and RNA in phase separated P-bodies, and thus likely enable P-body formation by liquid–liquid phase separation (LLPS). Supporting this, computational predictions suggested that NBDY can promote LLPS.^20^ In addition, in vitro studies demonstrated that NBDY mixed with RNA, but not NBDY alone, undergoes liquid–liquid phase separation (LLPS) by complex coacervation, forming droplets that depend on protein concentration and ionic strength.^50^ Phosphorylation of NBDY perturbed its droplet-forming behavior with RNA, consistent with a model in which this post-translational modification (PTM) shifts the charge balance and drives dissociation/remixing of the condensate. Overall, the data suggest that NBDY acts as a regulatory “switch.” However, several questions remain. It is not yet understood whether NBDY interacts with RNA directly or colocalizes with RNA via other protein binding partners in cells. Furthermore, the stoichiometry of NBDY within P-bodies and how its PTMs impact its interactions with other P-body proteins are also not fully resolved. Future studies are likely to uncover more depth on how NBDY mediates P-body LLPS.

### p14^ARF^

p14^ARF^ is a 132 amino acid microprotein encoded on a transcript isoform of *CDKN2A* that uses an alternative exon 1 but a shared exon 2. Translation of this isoform occurs in a different reading frame than *CDKN2A*’s other encoded protein, p16^INK4a^, thus producing a protein with an entirely different sequence.^51^ p14^ARF^ contains multiple arginine motifs that make it highly basic overall and enable its localization to the granular component (GC) of nucleoli, where it interacts with NPM1 under basal conditions.^52^ NPM1 is a nucleolar chaperone that organizes the granular component of the nucleolus via multivalent interactions between its acidic tracts and arginine-rich motifs in nucleolar proteins like p14^ARF^.^53^ p14^ARF^ is largely disordered and has been shown to undergo LLPS with NPM1 to form condensates in vitro*.* ^54^ A more recent study also reported that p14^ARF^ forms gel-like mesoscale assemblies driven by hydrophobic “sticker” residues in a partially folded N-terminal region, and NMR and small-angle neutron scattering (SANS) analyses support the emergence of α-helix and β-strand features within the ARF N-terminus in the condensed phase.^52^ Thus, p14^ARF^ is able to adopt some structural features upon binding like many other intrinsically disordered proteins.^55^

The tumor suppressor p53 plays a central role in responding to cellular stress, coordinating cell-cycle arrest, senescence, and apoptosis. Under basal conditions, p53 levels are kept low by the E3 ubiquitin ligase MDM2, which ubiquitinates p53 and targets it for proteasomal degradation. p14^ARF^ functions as a critical upstream sensor of oncogenic stress and DNA damage, which trigger its release from NPM1 and translocation into the nucleoplasm where it inhibits MDM2 to stabilize p53.^56^ Biophysical studies have demonstrated that the key interaction domains of p14^ARF^ and MDM2 are intrinsically disordered in isolation, but undergo a disorder-to-order transition upon binding, similar to what is observed for NPM1.^57^ This interaction is mediated by two short conserved RxFxV motifs located in the N-terminus of p14^ARF^, which bind two complementary segments within the central acidic domain of MDM2.^57,58^ In addition, short peptides encompassing the ARF N-terminal motifs were found to be sufficient for inducing structural formation upon binding to MDM2.^57^ These studies demonstrate how alternative small, disordered proteins can serve as key regulators of cellular pathways.

### Nucleolar Integrity and Stress Microprotein (NISM)

NISM is a recently discovered 36 amino acid aginine-rich microprotein that is essential for nucleolar formation and regulates rRNA synthesis.^59^ NISM was identified in HEK293T, K562, HeLaS3 and U2OS cells by Ribo-seq and hypothesized to have a role in RNA metabolism based on the presence of arginine-rich motifs. Computational analyses by AIUPred and ESMFold predicted NISM to be entirely disordered, and conformational ensemble modeling predicted it to be relatively rigid along its central arginine-rich region. Overexpression of NISM in U2OS cells induced shrinking and rounding of nucleoli accompanied by p53 stabilization, G2/M phase accumulation, impaired rRNA synthesis, and reduction of nucleolar R-loops. NISM knockout, on the other hand, caused disruption of nucleolar structure, which triggered p53 stabilization and G1/S phase accumulation. Importantly, nucleolar structure was restored in the knockouts upon rescue with NISM, demonstrating its role in LLPS-driven formation of nucleoli. Immunoprecipitation mass spectrometry (IP-MS) studies revealed that NISM interacts with the RNA helicase DHX9. In addition, DHX9, but not NISM, was predicted to promote LLPS, suggesting that DHX9 may be mediating NISM’s role in nucleolar formation. Supporting this, sequence charge decoration matrix mathematical modeling showed that NISM complexation with DHX9 increases DHX9’s propensity for LLPS. While computational tools for predicting disorder and polymer physics-based models of LLPS are useful, NISM’s structure and effects on DHX9 LLPS remain to be validated empirically.

Collectively, NBDY, p14^ARF^, and NISM demonstrate how disordered microproteins can regulate BMC formation through multivalent interactions that drive LLPS. Further, they illustrate how these activities can be characterized through a combination of computational modeling as well as biochemical, biophysical, and cell biological assays.

## Disordered Microprotein Interactors of Transmembrane Proteins

### Phospholamban (PLN)

PLN is a 52 amino acid sarcoplasmic reticulum membrane-embedded microprotein that serves as a critical regulator of the sarco­(endo)­plasmic reticulum Ca^2+^-ATPase (SERCA) in cardiac muscle.^60^ PLN belongs to a broader family of SERCA-regulatory microproteins known as “regulins.” While multiple regulins function in muscle cells, such as PLN, sarcolipin (SLN), and myoregulin (MLN), recent discoveries have expanded this family to include endoregulin (ELN) and another-regulin (ALN) which also function in endothelial and epithelial cells.^61^ PLN regulates SERCA’s calcium transporter activity and cardiac contractility by extension. Structurally, PLN comprises three distinct domains: a cytoplasmic domain Ia (residues 1–16), a connecting loop (residues 17–21), and a transmembrane region divided into domain Ib (residues 22–30) and domain II (residues 31–52).^62,63^ PLN’s cytoplasmic regulatory domain exists in a dynamic equilibrium between three structural states, a ground T state that is helical and membrane-associated, an excited R state that is unfolded and detached from the membrane, and a B state that is extended and bound to SERCA.^62,64,65^ The T and R states are inhibitory (low SERCA activity), while the B state is noninhibitory (high SERCA activity). Phospholamban (PLN/PLB) serves as an example for membrane-anchored microproteins that utilize intrinsic disorder to mediate function. While early structural models depicted PLN as a static “L-shaped” helix, extensive biophysical characterizations including NMR spectroscopy, electron paramagnetic resonance (EPR) spectroscopy, and electron microscopy, have since shown it to be a highly dynamic molecule with a functional IDR in its cytoplasmic domain and flexible loop.^62–65^ These disordered regions determine PLN’s inhibitory and noninhibitory states.

PLN has also been shown to interact with HAX-1 in cardiac muscle cells to regulate SERCA activity.^66,67^ Solid-state NMR experiments showed that HAX-1 binds the cytoplasmic region of monomeric PLN in lipid bilayers, while FRET measurements demonstrate interaction with PLN pentamers.^67^ Mapping studies identified residues 203–245 of HAX-1 as the minimal binding region targeting residues 16–22 of PLN.^66^ Magic angle spinning (MAS) solid-state NMR data further indicated that HAX-1 binding shifts PLN from a dynamic to a more rigid, and thus more inhibitory, conformational state, reinforcing the idea that the cytoplasmic IDR of PLN acts as a versatile interaction module whose structure is dictated by binding context.^67^ Overall, the identification of PLN and the other regulin family members suggests that IDR-driven conformational switching may be a conserved mechanism for tuning intracellular Ca^2+^ dynamics across diverse tissues.^61,68^

### Small Human Mitochondrial ORF over SErine tRNA (SHMOOSE)

SHMOOSE is a 58 amino acid microprotein discovered through a mitochondria-wide association study (MiWAS) that linked mitochondrial DNA variation to Alzheimer’s disease (AD).^69^ Large-scale epidemiological genetic studies showed that the SHMOOSE D47N variant exhibited an increased risk of developing AD. SHMOOSE localizes to mitochondria and interacts with mitofilin (IMMT), an inner mitochondrial membrane protein and core component of the MICOS complex that organizes mitochondrial crista junctions. SHMOOSE binding to IMMT was observed in multiple cellular models, including neuronal cells. Treatment with exogenous SHMOOSE increased neural cell metabolic activity and ICV treatment in rats resulted in altered expression of genes related to neuronal, immune, and mitochondrial processes. In addition, correlations between SHMOOSE levels in cerebrospinal fluid and Alzheimer’s disease-related biomarkers raise the possibility that SHMOOSE may serve as a mitochondrial biomarker for AD.

Structural prediction by RosettaFold and HeliQuest suggested that SHMOOSE contains a short N-terminal amphipathic α-helix, followed by a highly positively charged central region. The sequence following the N-terminal helix was predicted to be intrinsically disordered by IUPred3, although direct biophysical validation of disorder has not yet been reported. Future studies will be necessary to understand what role the disordered region of SHMOOSE has in regulating mitochondrial biology.

### Noxa

Noxa is a 54 amino acid pro-apoptotic microprotein encoded on the *PMAIP1* gene, and its expression is induced by p53 in response to DNA damage.^70^ Like CYREN, Noxa has multiple isoforms, including a 70 amino-acid and a 136 amino-acid variant that are also regulated by p53 but do not possess pro-apoptotic function. Noxa’s pro-apoptotic function is mediated by a BH3 domain, or SLiM,^71^ that binds the pro-survival outer mitochondrial membrane protein MCL-1.

As is typical for BH3 motifs, this segment is intrinsically disordered in isolation and folds into an α-helix only upon target engagement.^72^ Consistent with this observation, multiple studies classify Noxa as an intrinsically disordered protein based on computational predictions, thermal denaturation assays, and CD analysis.^73,74^ Furthermore, another study used electron paramagnetic resonance (EPR) spectroscopy of spin-labeled Noxa to show that that the BH3 region exists in two backbone conformations, a dynamically disordered R state and a helically ordered T state.^75^ Upon the addition of MCL-1, the EPR spectra showed a reduction in the population of the R state relative to the T state, and quantitative analysis revealed that the order parameter (S) and correlation time of the T state increased markedly. These changes indicated increased structural order and reduced backbone mobility, consistent with stable engagement of the BH3 motif by MCL-1.

Together, Noxa and PLN illustrate how disordered microproteins can serve as dynamic regulators of membrane proteins by switching between disordered and partially structured regions depending on their state. We predict that future studies of SHMOOSE’s disordered region may reveal that it similarly acts in a regulatory capacity.

## Regulation of Disordered Microprotein Interactions by Phosphorylation

IDRs’ lack of structure make them frequent targets of PTMs in part because they are highly accessible to modifying enzymes, such as kinases and phosphatases.^76^ PTMs within disordered regions can alter local charge distribution, disrupt or promote transient secondary structure, or modulate binding motifs, thereby shifting the conformational ensemble of the protein. As such, PTMs often function as molecular switches that regulate protein–protein interactions (PPIs), conformational equilibria, and higher-order assembly processes such as biomolecular condensate formation. While there are only a few examples thus far, disordered microproteins have been observed to exhibit phosphorylation-dependent regulation like many canonical IDPs and IDRs.

As described above, NBDY’s ability to form droplets with RNA in vitro is regulated by phosphorylation.^50^ Phosphoproteomics and biochemical assays revealed that NBDY is phosphorylated at Thr40 at the G2/M checkpoint downstream of CDKs and at Ser61 in response to EGFR signaling downstream of PKC. Expression of nonphosphorylatable mutants was found to impair normal disassembly of P-bodies during mitosis and EGFR signaling in cells, as was observed for the knockout (Figure 3A). In addition, the nonphosphorylatable mutants of NBDY were also unable to rescue the impaired proliferation effect observed in NBDY knockout cells. Thus, phosphorylation is a critical regulator of NBDY’s P-body disassembly activity.

**Figure 3:**
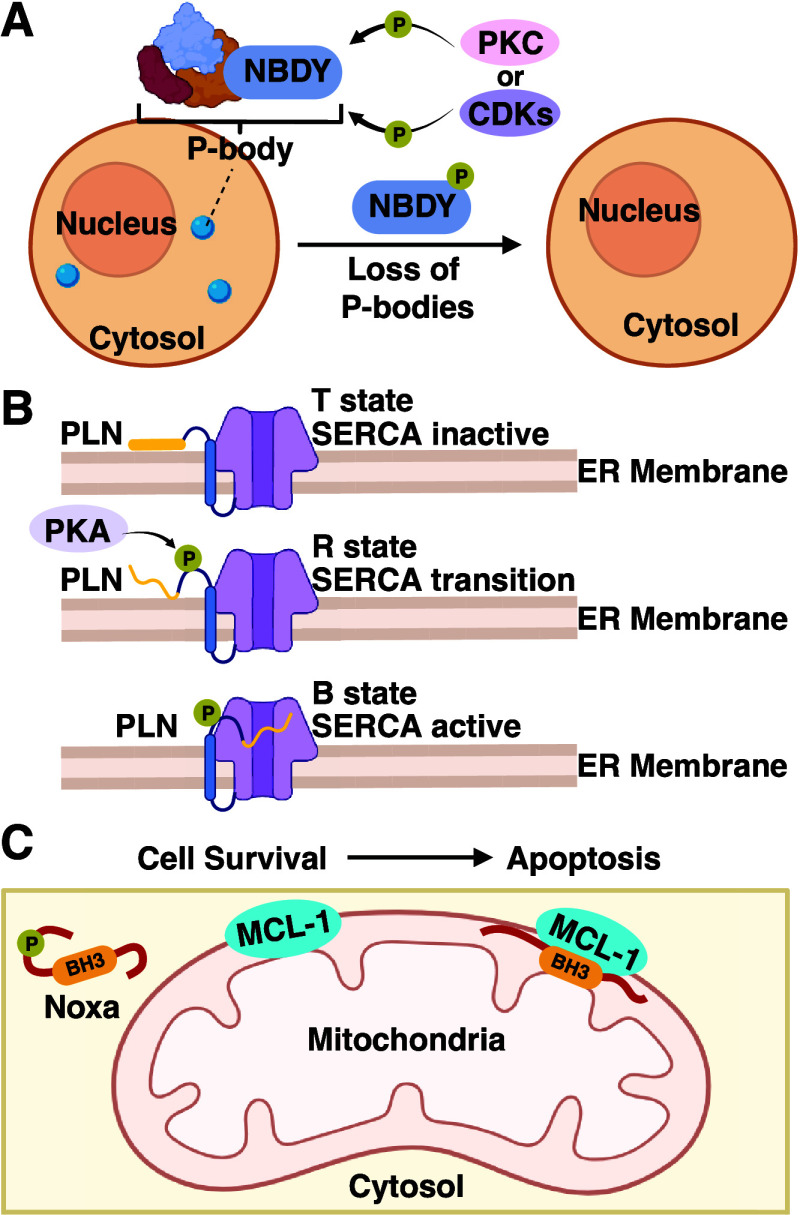
Phosphorylation can regulate the activity disordered microproteins. (A) Phosphorylation of NBDY at Thr40 or Ser61 downstream of EGFR signaling promotes P-body disassembly. (B) Phosphorylation of PLN at Ser16 or Thr17 induces a conformational change in the cytoplasmic domain that allows it to bind SERCA and relieve its inhibitory effect on SERCA. (**C**) Phosphorylation of Noxa at Ser13 prevents its BH3 domain from interacting with MCL-1 and activating apoptosis. Figure created using BioRender.com.

PLN’s shift between inhibitory and noninhibitory states is also highly dependent on its phosphorylation.^62–64,77^ PLN is phosphorylated at Ser16 by PKA and at Thr17 by CamKII.^17^ Unphosphorylated PLN inhibits SERCA, while phosphorylation at these sites promotes the PLN’s shift to the noninhibitory B state (Figure 3B). Numerous biophysical studies have demonstrated the impact of phosphorylation on PLN’s states. For instance, refocused insensitive nuclei enhanced by polarization transfer (rINEPT) ssNMR experiments demonstrated that phosphorylation induces an order-to-disorder transition in domain Ia when PLN is in its free form, destabilizing the membrane-associated T state and increasing the population of the R state.^62^ However, rINEPT and dipolar-assisted rotational resonance (DARR) experiments also showed that the phosphorylated cytoplasmic domain undergoes a disorder-to-order transition upon binding SERCA and adopts the B state conformation. Earlier solution NMR and molecular dynamics (MD) simulations found similar dynamic changes in PLN’s structure upon phosphorylation in the absence of SERCA.^78,63^ Notably, phosphorylation has minimal impact on the dynamics or orientation of the transmembrane domain, indicating that relief of inhibition is driven almost entirely by structural shifts in the disordered cytoplasmic region.^77^ These studies demonstrate the importance of phosphorylation on PLN’s structure and activity.

Like NBDY and PLN, Noxa is also regulated by phosphorylation. In proliferating leukemia cells, CDK5 phosphorylates Noxa at Ser13 in a glucose-dependent manner and inhibits its ability to bind MCL-1 and activate apoptosis^74^ (Figure 3C). Phosphorylated Noxa remains cytosolic and inactive, whereas glucose withdrawal triggers dephosphorylation and restores apoptosis. As detailed above, Noxa switches between two dynamic states and shifts toward the more ordered T state upon binding to MCL-1. However, EPR spectroscopy showed that unphosphorylated Noxa and phosphorylated Noxa are nearly identical, indicating that phosphorylation does not substantially alter the intrinsic T–R equilibrium of the BH3 motif itself.^75^ Instead, phosphorylation regulates Noxa through long-range, electrostatic interactions between phosphorylated Ser13 and Arg30, which in turn facilitates an interaction between Noxa’s N-terminus and BH3 domain. These observations help explain why phosphorylated Noxa is unable to bind MCL-1. Further supporting this, phosphorylated Noxa exhibits no detectable spectral changes upon addition of MCL-1. These results demonstrate how phosphorylation of disordered regions can also be used to restrict a proteins structure and prevent interactions with other proteins.

Altogether, these examples illustrate how phosphorylation can regulate disordered microproteins by reshaping their conformational ensembles. We expect future studies to uncover functional PTMs in additional disordered microproteins as well as PTMs beyond phosphorylation, such as acetylation, methylation, and glycosylation.

## Therapeutic Targeting of Intrinsically Disordered Regions

Microproteins are being investigated in a variety of disease contexts,^79^ particularly in different cancers.^80^ As such, microproteins found to be key regulators of disease progression could in theory serve as valuable therapeutic targets. However, largely disordered microproteins pose major barriers to therapeutic development. IDRs have long been considered challenging therapeutic targets because they lack stable pockets for conventional small-molecule binding.^81,82^ Their adoption of conformational ensembles, rather than a single defined structure, make it difficult to target any potentially more structured state, if such a state even exists. Furthermore, many disordered proteins interact with multiple protein binding partners, thus making it difficult to target only a specific disease-relevant interaction. Notwithstanding these issues, recent examples of targeting IDRs in canonical proteins have emerged that may be applicable to disordered microproteins.

Two recent examples of therapeutic approaches to target IDRs come from efforts to drug transcription factors (TFs). TFs are DNA binding proteins that regulate gene expression and frequently contain large, disordered regions.^83^ For instance, many TFs activate gene expression through intrinsically disordered transcriptional activation domains (TADs) that bind coactivator proteins.^84–87^ TADs exhibit structural plasticity, which allows them to engage multiple partners and regulate diverse signaling pathways, but also makes therapeutic targeting difficult. One recently developed strategy employs an engineered protein to target the TF, or other proteins of interest, and induce its ubiquitination and subsequent degradation by the proteasome.^88,89^ Inspired by the development of Proteolysis Targeting Chimeras (PROTACs), this approach fuses a highly specific IDR-binding nanobody to an E3 ligase adaptor and a cell-permeant mini-protein (Figure 4A). This nanobody-based degrader was originally developed to target BCL11A, a critical repressor of fetal globin gene transcription and target for the treatment of hemoglobinopathies.^88,90^ Importantly, their engineered protein needed to target BCL11A but not its highly similar paralog BCL11B. To achieve this, yeast surface display was used to select for a nanobody that recognizes the ordered and conserved zinc finger domains 2 and 3 as well as the immediate unstructured region following these domains where the two paralogs diverge in sequence. By then fusing this nanobody sequence to the cell-permeant mini-protein ZF5.3 and the E3 ligase adaptor tSPOP, this engineered protein was able to enter cells and degrade endogenous BCL11A specifically, resulting in significant induction of fetal hemoglobin in primary erythroid precursor cells.^88^ This approach represents a highly flexible approach for targeting IDRs, and proteins otherwise difficult to target with small molecules, for degradation.

**Figure 4:**
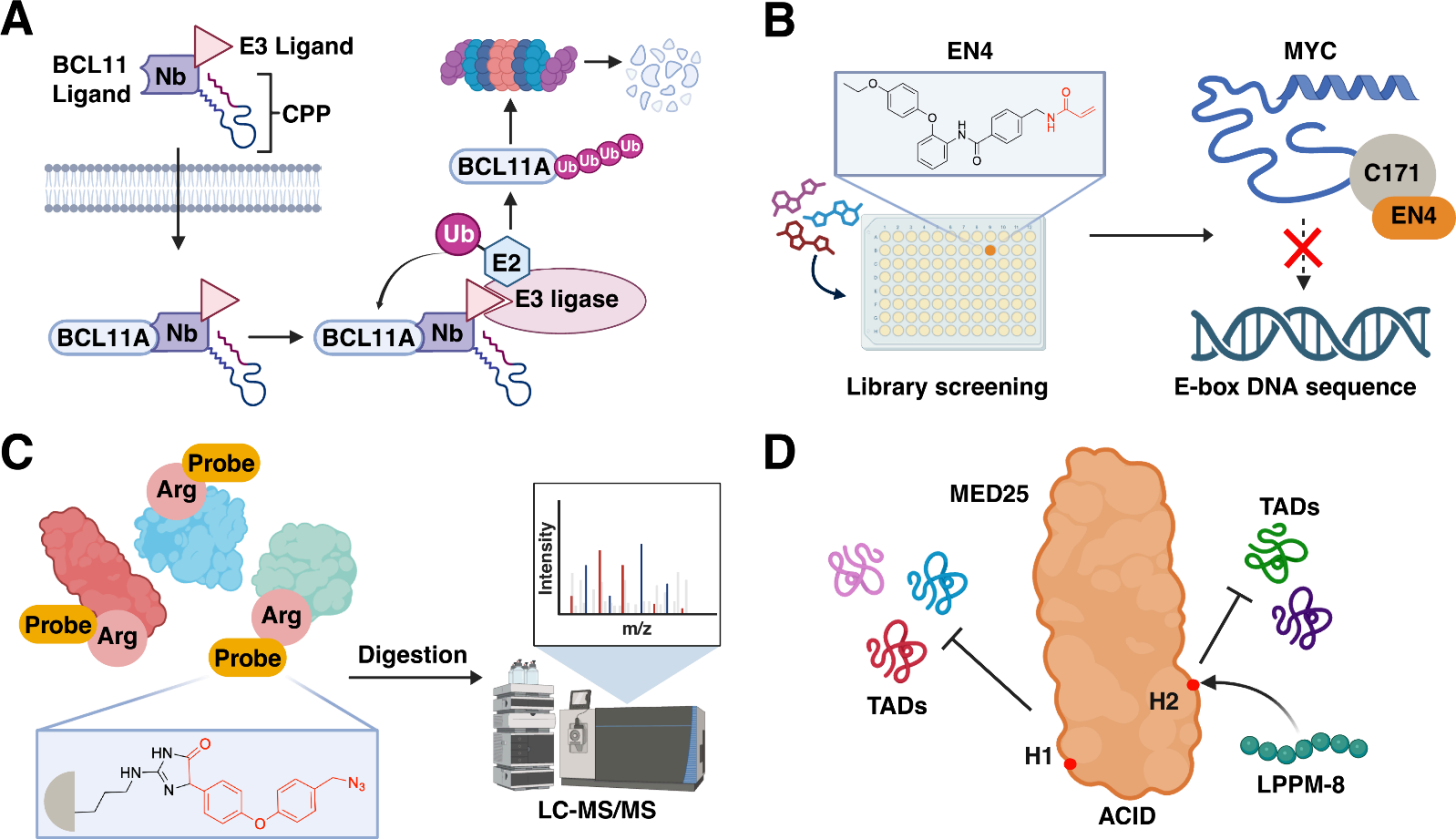
Strategies for targeting intrinsically disordered regions (IDRs). (A) Depiction of the nanobody-based degrader approach to induce ubiquitination and subsequent degradation of BCL11A. The engineered protein is composed of a nanobody that is selective for a disordered region of BCL11A, a cell-permeant mini-protein, and the E3 ligase adaptor tSPOP. (B) Depiction of small molecule library screening approach to identify covalent binders of reactive cysteines in IDRs. EN4 covalently modifies Cys171 within an intrinsically disordered region of MYC, destabilizing the MYC/MAX complex and suppressing MYC-dependent transcription. (C) Depiction of a chemoproteomic approach to identify ligandable arginine residues within IDRs that can be targeted to disrupt PPIs and modulate LLPS. (D) Depiction of how the lipopeptidomimetic LPPM-8 binds the highly dynamic H2 surface of MED25, inducing conformational changes that competitively inhibit H2-binding activators and allosterically inhibit H1 interactions. CPP, cell permeant protein. Ub, ubiquitin. Nb, nanobody. TADs, transcriptional activation domains. ACID, activator interaction domain. Figure created using BioRender.com.

A second approach to target TFs was developed to inhibit the oncogenic transcription factor MYC. MYC activates transcription through heterodimerization with MAX and binding to E-box DNA sequences. Direct targeting of MYC has long been considered difficult due to its highly disordered structure.^91^ To address this, a library of cysteine-reactive covalent ligands was screened to identify compounds capable of disrupting MYC/MAX DNA binding.^92^ This effort led to the discovery of EN4, an acrylamide-containing covalent ligand that inhibits MYC/MAX binding to DNA and suppresses MYC-dependent transcription in cells (Figure 4B). Proteomic analysis revealed that EN4 covalently modifies Cys171, a residue located within a predicted intrinsically disordered region of MYC. Isotopic Tandem Orthogonal Proteolysis-Activity-Based Protein Profiling (IsoTOP-ABPP) demonstrated that EN4 was highly selective, with only 8 off-target proteins among more than 1400 probe-labeled peptides. Among these off-target hits was the transcription factor JUN, which reacted with EN4 at Cys99. Despite this, EN4 retained comparable inhibitory effects in MYC luciferase reporter assays even when JUN was knocked down, indicating that JUN modification is unlikely to drive the compound’s suppression of MYC transcriptional activity. This study demonstrates that covalent ligand discovery coupled with chemoproteomics can reveal ligandable sites within intrinsically disordered regions and enable pharmacological targeting of proteins previously considered intractable.

In addition to targeting nucleophilic residues like cysteine, ABPP has also been used to map functionally important residues like arginine on a proteome-wide scale. Ligandable arginines are likely to participate in PPIs and those found within IDRs can help mediate LLPS.^93^ A recent study systematically profiled arginine reactivity in human cells using dicarbonyl-based probes and high-resolution mass spectrometry, quantifying more than 2,200 reactive arginine sites in a single cell line^94^ (Figure 4C). The covalently labeled arginines were predominantly surface-exposed and frequently engaged in cation−π interactions with aromatic residues, a typical interaction mode of IDRs. Notably, this study identified arginine residues that play key regulatory roles in LLPS in both the cytoplasm and nucleus. For instance, they showed that the R313K NONO mutant had reduced nuclear paraspeckle puncta size, while the R313A mutant showed reductions in both puncta size and number.^94^ Excitingly, most liganded arginines were found to be targeted by a single fragment probe, suggesting that this platform could be used to develop arginine-targeted covalent drugs to modulate phase separation and other IDR-mediated interactions.

Peptides designed to mimic interactions with IDRs represent another a potential strategy for targeting disordered microproteins. This approach has been demonstrated recently for Mediator complex subunit 25 (MED25), a component of the transcriptional coactivator Mediator complex.^95,96^ MED25′s activator interaction domain (AcID) recognizes diverse transcription factors through two hydrophobic binding surfaces, termed H1 and H2.^97,98^ Although different activators bind MED25 with similar affinities, each forms a conformationally distinct complex involving binding-coupled structural rearrangements within the AcID.^98^ The lipopeptidomimetic MED25 inhibitor LPPM-8 was developed to overcome this challenge^99^ (Figure 4D). LPPM-8 consists of a 7 amino acid amphipathic peptide based on sequences that frequently occur in MED25-interacting TADs and an N-terminal branched fatty acid. The addition of the branched fatty acid chain resulted in >20-fold increase in potency against MED25-activator PPIs while also maintaining >6-fold selectivity for MED25 versus other coactivator-activator complexes in vitro. Biophysical data indicated that LPPM-8 binds directly to the H2 face of Med25 AcID and induces conformational changes in the domain. Consequently, LPPM-8 acts as a competitive inhibitor of transcriptional activators that bind the H2 surface, such as ATF6α, and is predicted to allosterically inhibit activators that engage the H1 face. NMR data also indicated that LPPM-8 engages multiple dynamic substructures within Med25 AcID rather than a single rigid site, further suggesting that it is likely to be highly selective in cells.^99,100^ This work provides compelling support that IDR-mediated PPIs can be inhibited by peptidomimetics that target dynamic interaction landscapes.

These examples highlight the diversity of recently developed modalities and strategies for targeting IDRs. Based on the activities and mechanisms of disordered microproteins that have been functionally characterized thus far, we anticipate that these approaches will translate well to targeting microproteins.

## Conclusion

As the field continues to refine microprotein annotations across multiple species and functionally characterize individual microproteins, the overlap between microproteins and canonical IDPs has become more apparent. From the examples reviewed here, clear themes have emerged on how disordered microproteins function similarly to larger IDPs in different processes and cellular compartments. First, disordered microproteins often exert their activities by binding to and regulating larger proteins, both through competitive inhibition and activation. Second, disordered microproteins can promote complex formation by engaging with multiple binding partners. Third, disordered microproteins can also regulate the formation of different BMCs by LLPS. Finally, phosphorylation, and likely other PTMs, can help modulate the dynamic nature of disordered microproteins. Awareness of these paradigms can help researchers more quickly characterize microproteins with predicted disordered sequences.

These characterized disordered microproteins also provide a roadmap for how to explore their biophysical properties. The dynamic nature of disordered microproteins and IDPs requires methods that can capture the totality of their conformational ensembles in solution. To this end, these studies highlight the effectiveness of applying different spectroscopy methods, such as NMR and EPR. CD has also repeatedly been shown to be effective for determining whether any secondary structure can form throughout disordered microproteins. Similarly, HDX-MS is effective for identifying solvent exposed and unstructured regions of microproteins. Additionally, small-angle scattering approaches like SANS are effective for assessing the ensemble properties of disordered proteins, such as their radius of gyration, and proteomics is critical for unbiased detection of PTMs. While essential, empirical approaches to investigate disordered structures require significant expertise and equipment that may not be accessible. Therefore, computational tools for predicting disordered regions, such as IUPred,^101^ and modeling conformational ensembles using MD simulations or deep learning-based tools like STARLING^21^ can provide important first insights that may allow researchers to circumvent empirical approaches or inspire more focused biophysical experiments. As more disordered microproteins are characterized biophysically using these approaches, we foresee patterns arising in their molecular mechanisms that will allow for classification of subgroups of microproteins.

The majority of functionally characterized disordered microproteins reviewed here are evolutionarily conserved across other mammals. However, most microproteins are evolutionarily young.^102^ Thus, we expect that future efforts will uncover more examples of human- and primate-specific functional disordered microproteins. Indeed, a recent study used high-throughput protein interaction screen on peptide matrix (PRISMA) to identify dozens of protein interactions between canonical proteins and SLiMs located within predicted disordered regions of young microproteins.^102^ One such microprotein encoded on *LINC01128* was found to interact with clathrin heavy- and light-chain proteins and regulate intracellular trafficking and endocytosis. This study demonstrated the benefit of employing in vitro high-throughput interaction screens for uncovering disordered microproteins binding partners. We believe that PRISMA and similar approaches, such as proteomic peptide-phage display (ProP-PD),^103^ will dramatically expand the number of functional disordered microproteins discovered.

In closing, we appear to be just scratching the surface in terms of understanding the importance of disordered microproteins in cell biology and physiology. Therefore, continued collaboration across the microprotein and IDP fields will be indispensable for developing new approaches to study these unique proteins and expand our appreciation for how a lack of structure can often be more effective than a defined structure for some functions. Similarly, working across disciplines with chemical biologists and medicinal chemists will be essential for overcoming the challenge of developing therapeutics to target disordered microproteins in disease.
